# Patients With Common Variable Immunodeficiency (CVID) Show Higher Gut Bacterial Diversity and Levels of Low-Abundance Genes Than the Healthy Housemates

**DOI:** 10.3389/fimmu.2021.671239

**Published:** 2021-05-14

**Authors:** Juraj Bosák, Matej Lexa, Kristýna Fiedorová, Darshak C. Gadara, Lenka Micenková, Zdenek Spacil, Jiří Litzman, Tomáš Freiberger, David Šmajs

**Affiliations:** ^1^ Department of Biology, Faculty of Medicine, Masaryk University, Brno, Czechia; ^2^ Faculty of Informatics, Masaryk University, Brno, Czechia; ^3^ Centre for Cardiovascular Surgery and Transplantation, Brno, Czechia; ^4^ Department of Clinical Immunology and Allergology, Faculty of Medicine, Masaryk University, Brno, Czechia; ^5^ RECETOX Center, Faculty of Science, Masaryk University, Brno, Czechia; ^6^ Department of Clinical Immunology and Allergology, St. Anne’s University Hospital in Brno, Brno, Czechia

**Keywords:** common variable immunodeficiency, CVID, microbiome, metagenome, metabolome, *Hungatella hathewayi*

## Abstract

Common variable immunodeficiency (CVID) is a clinically and genetically heterogeneous disorder with inadequate antibody responses and low levels of immunoglobulins including IgA that is involved in the maintenance of the intestinal homeostasis. In this study, we analyzed the taxonomical and functional metagenome of the fecal microbiota and stool metabolome in a cohort of six CVID patients without gastroenterological symptomatology and their healthy housemates. The fecal microbiome of CVID patients contained higher numbers of bacterial species and altered abundance of thirty-four species. *Hungatella hathewayi* was frequent in CVID microbiome and absent in controls. Moreover, the CVID metagenome was enriched for low-abundance genes likely encoding nonessential functions, such as bacterial motility and metabolism of aromatic compounds. Metabolomics revealed dysregulation in several metabolic pathways, mostly associated with decreased levels of adenosine in CVID patients. Identified features have been consistently associated with CVID diagnosis across the patients with various immunological characteristics, length of treatment, and age. Taken together, this initial study revealed expansion of bacterial diversity in the host immunodeficient conditions and suggested several bacterial species and metabolites, which have potential to be diagnostic and/or prognostic CVID markers in the future.

## Introduction

The human gut microbiome is a complex microbial ecosystem significantly contributing to the host physiology. A number of factors including host genetics, age, diet, and antibiotic exposure can change the composition of the gut microbiota. Microbial dysbioses have been associated with various metabolic (obesity, diabetes) and intestinal (inflammatory bowel diseases, colorectal cancer) diseases, with diseases of the cardiovascular system, liver or kidney, and also with psychiatric disorders. Since the gut microbiota modulates the host immune system, a microbial dysbiosis was found in immune conditions including allergy, asthma, atherosclerosis, and multiple sclerosis. The gut microbiome and its role in human health and disease has been recently extensively reviewed (1–4).

Common variable immunodeficiency (CVID) is the most common primary immunodeficiency with a frequency of 1:25,000 to 1:50,000 in the human population worldwide. CVID includes clinically and genetically heterogeneous disorders characterized by a defect in B cell differentiation leading to inadequate antibody responses and low levels of immunoglobulin G (IgG) and IgA, and, inconsistently, also IgM. As a result of this deficient antibody production, most patients suffer from recurrent respiratory and gastrointestinal infections and from noninfectious autoimmune or inflammatory complications. This, together with clinical heterogeneity, are the main reasons why CVID is frequently misdiagnosed and generally underdiagnosed. The current therapy is based on administration of IgG and, in some patients, immunosuppression, in order to manage the infectious and autoimmune complications, respectively [reviewed in (5–9)].

Since IgA is the dominant mucosal immunoglobulin maintaining intestinal homeostasis (10), CVID is expected to impact the gut microbiome. Indeed, several studies showed changes in bacterial composition of the gut associated with CVID and with severity of the disease (11–15). While previous studies analyzed CVID microbiome using sequencing of 16S rRNA gene, we performed metagenome deep-sequencing for identification of differences among bacterial species and genetic functions. Moreover, metagenome findings have been combined with analysis of stool metabolites. Effects of CVID complications (e.g., chronic diarrhea) and environment have been minimized by enrollment of a cohort of six CVID patients without gastroenterological symptomatology and their healthy housemates. This initial study revealed, for the first time, an expansion of gut bacterial diversity in the immunodeficient conditions and suggested several bacterial species and metabolites as potential markers for CVID. Since the differences associated with CVID have been identified across the patients with various immunological characteristics, various age, and various lengths of treatment, the identified metagenome and metabolome changes are relevant to diagnosis rather than particular symptoms or patients’ age.

## Materials and Methods

### Study Design, Recruitment of Participants, and Ethical Approval

This study extends our previous study, where 16S rRNA gene analysis was used for characterization of microbiome composition of CVID patients (13). Here, fecal microbiomes of CVID patients were characterized using metagenome sequencing of CVID patients and healthy partners sharing the same households. The use of paired samples minimalized influence of environmental factors on microbiome composition. Moreover, the effect of CVID-related complications on microbiome was eliminated by exclusion of patients with severe CVID phenotypes (e.g., enteropathy, chronic diarrhea) from this study.

The stool samples were collected between 2016 and 2017 from patients being treated at the St. Anne's University Hospital in Brno (Czech Republic) and their household members were used as paired healthy controls. All participants were Caucasians born and living in the Czech Republic. The healthy controls provided a self-report questionnaire and the patients fulfilled CVID-ICON diagnostic criteria (16). The pairs for this study were randomly selected from the set of 8 participants in our study Fiedorová et al. (13) after exclusion of immunosuppression treatment and CVID phenotypes with gastrointestinal complications.

Information about participants is shown in **Tables 1** and **S1**. All human clinical samples were collected after patients/healthy volunteers gave written informed consent regarding their participation in the study. All data used in the study were anonymized and the study was approved by the Ethic Committee of the Faculty of Medicine, Masaryk University (Protocol no. 37/2016).

**Table 1:** Clinical characteristics of the CVID patients.

Pair	Sample ID	Disease severity score (category)^a^	Leukocytes (10^9^/L)	Thrombocytes (10^9^/L)	Lymphocytes (10^9^/L)	Neutrophils (10^9^/L)	B cells (%)	B cell phenotype^b^	IgG (g/L)	IgA (g/L)	IgM (g/L)	Length of treatment (Years)	Chronic diarrhea	Bronchiectasis	Autoimmunity	Splenomegaly	Atrophic gastritis	Nodular hyperplasia
1	**PID003**	37 (more severe)	3.7	74	1.1	2.2	22.0	smB-21lo	8.59	<0.07	<0.08	21	No	Yes	Yes	Yes	No	No
2	**PID009**	6 (less severe)	5.6	236	1.3	3.6	2.6	smB-21norm	5.51	<0.07	<0.08	16	No	No	Yes	Yes	ND	ND
3	**PID012**	2 (less severe)	4.1	248	2.0	2.6	19.0	smB+ 21lo	12.5	0.319	<0.08	9	No	No	No	No	ND	ND
4	**PID004**	1 (less severe)	7.9	197	2.3	4.6	20.0	smB-21lo	6.01	<0.07	0.100	20	No	No	No	Yes	ND	ND
5	**PID036**	6 (less severe)	6.1	241	1.2	4.1	1.0	No B cells	7.46	<0.07	<0.08	6	No	No	Yes	No	ND	ND
6	**PID048**	1 (less severe)	4.3	233	1.1	2.7	11.0	smB-21norm	4.28	<0.07	0.164	1	No	No	No	Yes	ND	ND

^a,b^CVID was defined according to Ameratunga (6) and EUROclass (17), respectively.

ND, not determined.

For more characteristics of CVID patients and paired healthy housemates, see **Table S1**.

### Stool Collection and DNA Extraction

In this study, the genomic DNA was freshly isolated from the frozen stool aliquots originated from our previous study (13). Collection of stool samples and extraction of DNA were performed as described previously Fiedorová et al. (13). Briefly, stool samples were self-collected using a sterile container, according to the standardized International Human Microbiome Standards (IHMS) protocol SOP 03 and delivered to the laboratory in 24 hours; where were aliquoted (200 mg), frozen, and stored at -80°C. From fecal aliqoutes, DNA was extracted using the current standard operating procedure (protocol Q, International Human Microbiome Consortium) with minor modifications described in Fiedorová et al. (13). DNA eluates were stored at −20°C until processing.

### Metagenomic Sequencing

The preparation of DNA libraries and whole genome sequencing were performed in Novogene Co., Ltd. (Hong Kong) with requested 100 Gb data output per each sample. Briefly, after quality control of extracted DNA, 300 bp fragments were prepared by sonication and the DNA libraries were constructed using NEBNext® Ultra™ II DNA Library Prep (New England Biolabs, USA). The libraries were diluted to 2 ng/μL and their quality (>3 nM) was verified by qPCR. The libraries were sequenced using HiSeq platform (Illumina, USA) with paired-end strategy (150 bp).

On average, metagenomic sequencing resulted in 365,613,222 reads and 109.69×10^9^ bases per sample (**Table S2**).

The data are available under BioProject ID: PRJNA666684 (NCBI database; https://dataview.ncbi.nlm.nih.gov/object/PRJNA666684).

### Bioinformatic Analysis

#### Data Preparation and Primary Metagenomic Analysis

To ensure proportional analysis, a set of 160 million reads for each sample was randomly chosen and used for bioinformatic assembly. The raw sequencing data was checked for purity and quality using the FastQC software (18). Paired reads were subsequently assembled into contigs using Velvet (19) with k-mer size 31 and the -*shortPaired* read type setting. A metagenomic velvetg-meta postprocessing step was used as described by Afiahayati et al. (20), yielding a FASTA file with contigs for each sample. A magnitude variable representing read coverage was set in the FASTA header for use by downstream programs DIAMOND and MEGAN-LR. Contig count and size characteristics (maximal length, N50) were determined by running countN50.pl (Manapatra, downloaded Sep 4, 2018). Other statistics were obtained using common command-line tools or simple Perl and R scripts.

As a prerequisite for taxonomic and functional analysis, the assembled contigs were mapped to reference sequences from the *nr* database using DIAMOND software (21) using the following settings:

--range-culling --frameshift 15 --top 8 -p 15 -e 0.00001

The resulting *.*m8* files were then subjected to further statistical analysis and visualization.

#### Taxonomic and Functional Data Analysis

Further analysis was based mainly on counting and clustering alignments with MEGAN6-LR Community edition (22). All samples were analyzed against MEGAN taxonomy files (23) as well as SEED functional assignments (24) as recommended by authors of the software. Specifically, the longRead mode was chosen in the “Import BLAST and READs files” dialog and longReads with readMagnitude weights were chosen as LCA parameters for the binning/counting process. Minimal relative abundance to report was set to 0.02%. Counts were summarized for all subclasses and reported as relative or absolute counts for taxonomy and functional data. Neither raw reads, nor contigs were filtered for eukaryotic or viral sequences. In spite of this fact, corresponding taxa rarely passed the minimum reporting threshold (i.e., relative abundance <0.02%).

#### Alpha- and Beta-Diversity, Distance Measures, and Clustering Significance Permutation Tests

Alpha-diversity was calculated using the estimate_richness() function from the phyloseq R package (25) (**Supplemantary Methods**).

To assess beta-diversity in our samples and to evaluate how much of the inter-sample variability follows clustering by diagnosis and clustering by household (two main factors followed in the study), we employed the distance measures implemented by the vegan R package (26). The abundance tables (previous paragraph) were imported with the phyloseq R package (25) to create a valid biom data object. The functions ordinate() and plot() were then applied to this object with several vegan distance measures (i.e., Bray-Curtis, Chao, Gower, and Mountford) to generate NMDS ordination plots (see ordination.R script in **Supplementary Methods**). Vegan package distance() function using the same measures followed by hierarchical agglomerative clustering with hclust() was used to generate clustering trees (see clustering.R in **Supplementary Methods**).

To evaluate how much of the inter-sample variability follows clustering by diagnosis and clustering by household, we employed permutation tests implemented in the anosim() function of the vegan R package. If two groups of sampling units are really different in their microbial or functional composition, then compositional dissimilarities between the groups should be greater than those within the groups. These differences are tested for significance against differences in groups with random label permutations and assigned a p-value.

#### Differential Analysis for Diagnosis

Differential analysis between two groups of samples defined by diagnosis was carried out to see differentially distributed individual taxons and functions in addition to the global clustering patterns above.

R package DESeq2 (27) was used to identify differentially abundant taxons and functions. Abundance tables were processed with the deseq2.R script (**Supplementary Methods**).

R package ALDEx2 was used to create an effect plot that displays between-group differences in relation to respective underlying variability for every component of a high-dimensional dataset (28, 29). Abundance tables were processed using the aldex2.R script (**Supplementary Methods**).

### Metabolomic Analysis

Dried stool samples were extracted using 250 μl of 80% isopropanol. Each sample was mixed, centrifuged, and the supernatant was transferred into a new vial. A ten-fold diluted sample (2 µL) was injected (three replicates of each sample) on Orbitrap Fusion interfaced with the Shimadzu UHPLC system (Nexera X2, LC-30AD). Waters Acquity CSH column (100 x 2.1 mm, 1.7 μm) coupled with Aquity UPLC VanGaurd^TM^ C18 Precolumn (1.7 μm, 2.1 x 5mm) was used for the reverse phase separation. The column was thermostated at 30°C, and the flow rate was 0.3 ml/min. The mobile phase consisted of buffer A (0.5 mM ammonium fluoride) and buffer B (methanol). The gradient elution program was as follows: 0 min 2% B; 2 min 2% B; 11 min 95% B; 12.99 min 95% B; 13 to 19 min 2% B. Parameters of electrospray ionization in positive ion mode were as follows: sweep gas (Arb), 2; sheath gas (Arb), 30; auxiliary gas (Arb), 5; ion transfer tube temperature, 350°C; vaporizer temperature, 300°C and spray voltage, 4000 V. MS1 scan parameters: mass range, *m/z* of 100-1000; spectrum data type, centroid; resolution at 400 *m/z*, 60,000; maximum injection time, 50 ms; automated gain control (AGC) target, 40,000; lenses RF level, 60%. MS/MS was acquired for the targeted masses list with parameters: activation type, HCD; mass range, *m/z* of 50-1000; collision energy (%), 35; stepped collision energy (%), 5; resolution, 15,000; AGC target, 50,000; maximum injection time (ms), 100.

Raw data were processed using XCMS online and exported as a CSV file containing *m/z*, retention time, and peak area. Detected features were annotated in HMDB and KEGG databases for the putative identification of metabolites. MS/MS spectra were acquired for tentative identifications statistically different between CVID patients and control group, and fragments were matched against METLIN, MassBank, and mzCloud to confirm the structure. The peak area of each identified feature was normalized to the mean peak area. Generated area ratio was further divided by the fecal sample dry weight. Relative peak areas of all the metabolites were submitted for Quantitative enrichment analysis using MetaboAnalyst (version 4.0) to perform pathways visualization (30). Partial least squares discriminant analysis, implemented in MetaboAnalyst, was used for the association analysis of eight differential metabolites in CVID patients and controls.

### Statistical Analysis

A two-tailed nonparametric Mann-Whitney U test, Fisher’s exact test, and Wilcoxon paired test (Prism 5 software, GraphPad) was used for the analysis of statistical differences between two groups. Analysis of similarities (ANOSIM) and Partial least squares discriminant analysis (PLS-DA) was used for the identification of associations with CVID diagnosis. Statistical significance was considered on level p<0.05 and statistical trend on level 0.05<p<0.1. Multivariate analysis, i.e., Differential gene expression analysis based on the negative binomial distribution (DESeq2), Analysis of differential abundance taking sample variation into account (ALDEx2), and Quantitative enrichment analysis (QEA), were used for identification of differences in microbial, functional, and metabolic compositions between two study cohorts. P-values were adjusted for control of false discovery rate using Benjamin-Hochberg method. Statistical significance was considered on level p<0.05 and statistical trend on level 0.05<p<0.1.

## Results

The CVID patients enrolled into this study were non-smoking omnivores without chronic diarrhea or other accompanying gastrointestinal conditions, which could impact the microbiome composition. Five out of six CVID patients were mild cases with Ameratunga scores (6) of 6 or less, while a single case had a more severe manifestation. Cohort of patients contained various B cell phenotypes (i.e., smB+21lo, smB-21lo, no B cells, and smB-21norm) according to EUROclass (17). Serum IgA levels were undetectable (<0.07 g/l) in five patients, of which three had also undetectable IgM (<0.08 g/l). All patients have undergone a repeated IgG therapy. Within a year prior to stool collection, three patients were administered antibiotics, but not for at least a month prior to sampling (**Tables 1**; **S1**). The oldest patient PID003 (66 years, diagnosed for twenty years, repeated intravenous IgG treatment, Ameratunga score: 37, splenomegaly, bronchiectasis, and thrombocytopenia) also had the most severe symptoms.

To minimize the effects of environment and nutrition, the controls used in this study were healthy housemates of the CVID patients (**Table S1**). As a result, the paired sets contained six males and six females. The cohort of CVID patients contained four females and two males (p=0.567, Fisher’s exact test) with an average age of 44.3 years (ranging from 26 to 66 years). Healthy controls were four males and two females with an average age of 45.0 years (ranging from 25 to 67 years). Similarly to gender, there were no significant differences between patients and controls in age, height, weight, and body mass index (i.e., p=0.937, p=0.520, p=0.126, and p=0.149, respectively, Mann-Whitney test).

### The CVID Gut Microbiome Has Similar Total Numbers of Identified Unique Genes as the Microbiome of Healthy Housemates

**Figure 1:**
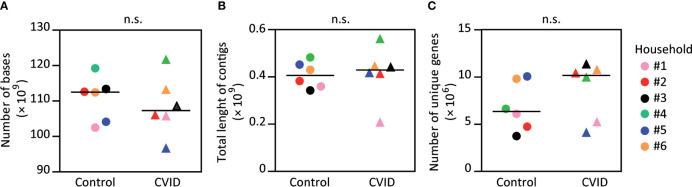
The fecal microbiomes of CVID patients and healthy controls have similar numbers of determined bases, contig lengths, and numbers of unique genes. The obtained metagenome data showed no difference in number of determined nucleotides **(A)** and the length of contigs **(B)** between the CVID patients and healthy controls. The numbers of unique genes were also similar **(C)**. Please, notice an insignificant increase of unique genes for CVID. Mann-Whitney test; n.s., not significant. Symbols, individuals. Black bar, median. Detailed characteristics of samples are shown in **Table S2**.

To study the effect of CVID on fecal microbiota, we performed a shotgun whole genome sequencing of stool samples obtained from the CVID patients and their healthy housemates. Obtained number of determined nucleotides and total length of contigs did not differ between the CVID patients and healthy controls (p=0.818 for both parameters, Mann-Whitney; **Figures 1A, B**; **Table S2**); and thus, the obtained sequencing data to be used for later analyses were of similar quality for both groups.

Subsequently, we analyzed the number of unique genes in each sample set using the *nr* database and DIAMOND sequence mapping software. The obtained values ranged from 3.73×10^6^ (PID011, control) to 11.35×10^6^ (PID012, CVID), and both extremes belonged to the same pair. Interestingly, the average number of unique genes was higher in CVID patients when compared to controls (8,624,337 and 6,839,204, respectively); however, this increase was not statistically significant (p=0.240, Mann-Whitney; **Figure 1C**; **Table S2**).

### CVID Fecal Microbiome Shows Increased Bacterial Diversity and Differences in Bacterial Species

**Figure 2:**
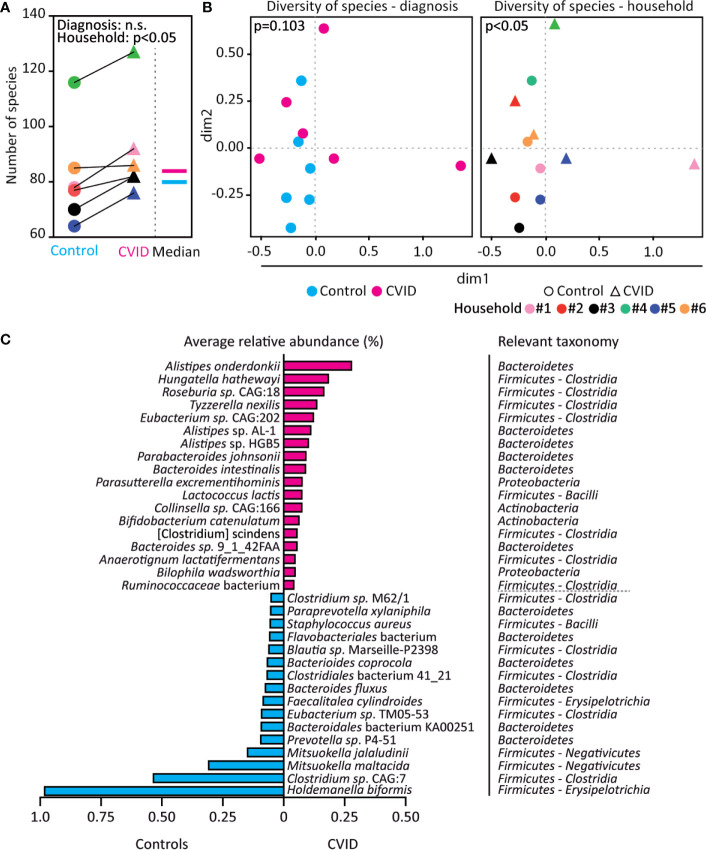
The CVID fecal microbiome shows increased bacterial diversity and differences in bacterial species. **(A, B)** CVID patients have more bacterial species than healthy housemates, irrespective of the total number of bacterial species identified in the corresponding CVID-control pairs. However, the microbiome composition also associates with household in addition to diagnosis. **(C)** Bacterial species with significantly increased (magenta) and decreased (cyan) relative abundance in CVID patients compared to healthy housemates (q<0.001, p-values adjusted for control of false discovery rate). Unpaired Mann-Whitney (for Diagnosis) and paired Wilcoxon tests (for Household) **(A)**, Chao dissimilarity matrix, NMDS plot, and nonparametric test ANOSIM **(B)**, and DESeq2 method **(C)** were used to calculate statistical differences. The complete analysis of taxonomic diversity, including a list of relative abundancies, is shown in **Figure S1**, **Table S3**, and **Table S4**.

To analyze the microbial composition of the stool samples, we matched the obtained metagenomes (i.e., assembled contigs) with the MEGAN taxonomy database using MEGAN-LR and read coverage reported by Velvet. While CVID patients and their housemates did not differ in numbers of genera and higher bacterial taxa (**Figure S1**), more bacterial species were found in CVID patients than in corresponding controls, which was observed in all six household pairs (p<0.05, Wilcoxon; **Figure 2A**). In addition, CVID patients showed a trend towards different composition of the bacterial species (p=0.103, ANOSIM (Analysis of similarities); **Figure 2B** left) resulting from differences in numbers rather than abundances of bacterial species (**Table S3**). As expected, we also found a significant effect of the environment and nutrition on the microbiome composition presented as clustering of the housemates (p<0.05, ANOSIM; **Figure 2B** right, **Table S3**).

A differential gene expression analysis based on the negative binomial distribution (DESeq2), which is suitable for metagenomic analysis of small sample sets (27), revealed that the CVID patients differed from healthy controls in the abundance of eleven genera belonging to *Firmicutes* and one genus from *Actinobacteria*. Specifically, the genera *Hungatella*, *Erysipelatoclostridium*, *Tyzzerella*, *Anaerotignum*, and *Anaeromassilibacillus* were more abundant in CVID patients, while *Mitsuokella*, *Megasphaera*, *Holdemanella*, *Acidaminococcus*, *Faecalitalea*, *Staphylococcus*, and *Actinomyces* were less abundant in CVID patients compared to healthy controls (q<0.001, DESeq2; **Table S4**). Furthermore, the relative abundance of a total 34 species differed between CVID patients and controls (q<0.001, DESeq2), with enrichment of 18 species and depletion of 16 species in CVID patients (**Figure 2C**). Interestingly, *Hungatella hathewayi* was frequent among CVID patients (in 5 out of 6 samples), but was not found among controls at all (**Table S4**).

### CVID Fecal Microbiome Shows Differences in Genetic Functions

**Figure 3:**
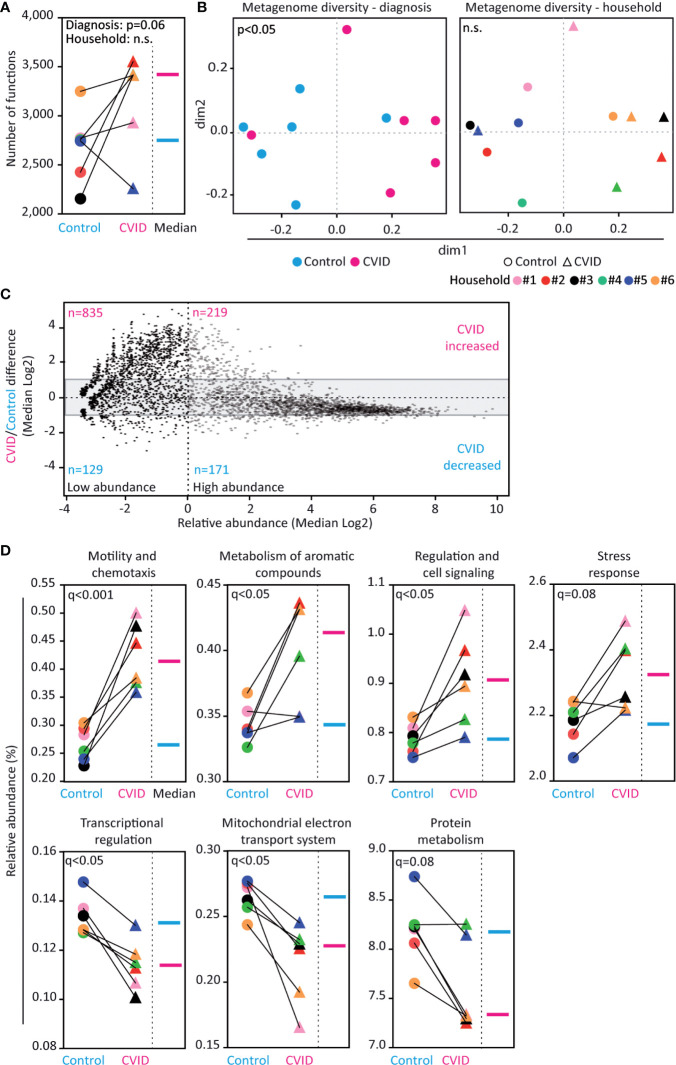
Fecal microbiome of CVID patients shows differences in genetic functions. **(A, B)** CVID patients have more database-identified genetic functions than healthy housemates (p=0.06). The functional metagenome associates with the diagnosis of CVID and not with household. **(C)** Composition of genes in the CVID metagenome differ from the metagenome of healthy controls. The CVID metagenome was enriched for low-abundance genes (see upper left corner). The grey area (-1 to +1) contains genes with similar abundance between groups (i.e., fold change lower than two). Based on the value of relative abundance 1 (dashed vertical line), the genes were classified as low-abundance (left dimension) and high-abundance (right dimension). **(D)** Relative abundance of functional groups within the metagenomes. Five functions showed significantly different abundance between the groups (q<0.05) and two other functions showed a corresponding trend (0.05<q<0.1). Unpaired Mann-Whitney (for Diagnosis) and paired Wilcoxon tests (for Household) **(A)**, Chao dissimilarity matrix, NMDS plot, and nonparametric test ANOSIM **(B)**, ALDeX2 method (Analysis of differential abundance taking sample variation into account) **(C)**, and DESeq2 method **(D)** were used to calculate the statistical differences. Database SEED level 3 **(A–C)** and level 1 **(D)** was used for functional analysis. The complete analysis of functional diversity, including a list of relative abundancies, is shown in **Figure S1**, **Table S3**, and **Table S5**.

In order to identify the composition of genetic functions in the CVID microbiome, we matched the obtained metagenomes with the SEED database (24). Altogether, 42% of the contigs were mapped to functional nodes in the database, while the remaining 58% of contigs were not found therein. Compared to controls, CVID patients showed a trend for increased functional richness (p=0.06, Mann-Whitney, **Figure 3A**) and, more importantly, a different composition of genetic functions (p<0.05, ANOSIM, **Figure 3B** left; Shannon index: p<0.05, Wilcoxon, **Table S3**). Specifically, the CVID metagenome contained enrichment of low-abundance genetic functions (i.e., median abundance <1%; **Figure 3C**). Unlike in microbial composition, the composition of functions was not associated with households (**Figure 3B** right; **Table S3**).

Among the 4,301 genetic functions with a hit in the SEED database, the relative abundances of 242 genes were significantly altered between CVID and control groups (q<0.05, DESeq2); most of the hits were genes enriched in CVID (**Table S5**). Moreover, CVID patients significantly differed from controls in the abundance of 38 pathways (q<0.05, DESeq2; **Table S5**). Specifically, the CVID metagenome was significantly enriched in the functions encompassing *Motility and chemotaxis* (q<0.001, DESeq2), *Metabolism of aromatic compounds* (q<0.05), and *Regulation and cell signaling* (q<0.05). Conversely, the functions *Transcriptional regulation* and *Mitochondrial electron transport system* were decreased (q<0.05, DESeq2; **Figure 3D**; **Table S5**) in CVID.

### CVID Metabolome Shows Dysregulated Purine Metabolism

**Figure 4:**
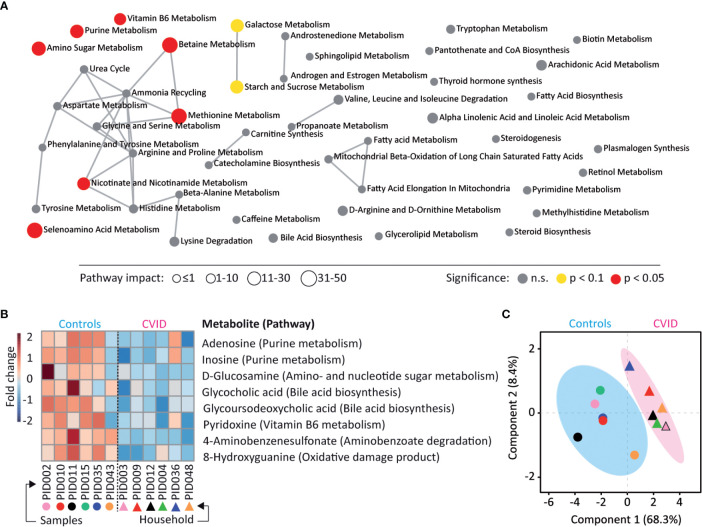
CVID stool metabolome shows differences in purine metabolism. **(A)** Metabolites identified in the LC-MS metabolome analysis belonged to 46 metabolic pathways and seven pathways showed dysregulation between CVID and healthy controls (p<0.05, QEA). Symbol size represents the pathway impact, which considers the relative abundance of metabolites in pathways (30, 31). **(B)** A heat map of eight metabolites significantly different between CVID and controls (p<0.05, Mann-Whitney). **(C)** The association of significant metabolites with diagnosis (R^2^=0.86, PLS-DA). **(A–C)** The MetaboAnalyst was used for visualizations and statistical calculations. Complete metabolomic analysis is shown in **Table S6**.

To identify metabolites possibly altered in the CVID gut, the stool samples were subjected to LC-MS untargeted metabolomics. In total 189 metabolites were identified and clustered into 46 metabolic pathways (**Table S6**). Seven of these metabolic pathways showed a significant dysregulation in CVID (p<0.05, Quantitative enrichment analysis (QEA), **Table S6**), involving metabolism of purines, amino sugars, selenoamino acids, methionine, betaine, nicotinate and nicotinamide, and vitamin B6 (**Figure 4A**). In addition, the level of eight individual metabolites differed significantly between CVID patients and controls (p<0.05, Mann-Whitney; **Figure 4B**). Specifically, the levels of adenosine, inosine, glucosamine, glycocholic acid, glycoursodeoxycholic acid, pyridoxine, 4-aminobenzenesulfonate, and 8-hydroxy guanine were lower in CVID than in controls. These metabolite changes tightly associated with CVID diagnosis (R^2^=0.86, Q^2^=0.34, Partial least squares discriminant analysis (PLS-DA); **Figure 4C**). Out of these metabolites, decreased adenosine and inosine levels represented purine metabolism, a function also decreased in the CVID metagenome (i.e., *De novo Purine biosynthesis*, q=0.08; **Table S5**).

## Discussion

While there are many studies focusing on the microbiome composition in various human conditions (1–4), few studies have analyzed the gut microbiome in CVID patients until now. While Jørgensen et al. (11), Fiedorová et al. (13), and van Schewick et al. (14) characterized the fecal microbiome of CVID patients based on analysis of 16S rRNA gene (44, 27, and 30 patients, respectively), Fadlallah et al. (15) performed a metagenomic analysis of 7 CVID patients. In addition, Shulzhenko et al. (12) analyzed microbiome from biopsies of 15 patients with various CVID severity and Mohammed et al. (32) analyzed the microbiome of a murine CVID model (i.e., CD19-/- mice). To our knowledge, our study is the first to combine the shotgun metagenomic and metabolomic approach for microbiome comparison of CVID patients with healthy controls living in the same household.

In this initial study, a cohort of six CVID patients and their healthy housemates was analyzed. The limited size of samples is a result of the relatively low prevalence and underdiagnosis of CVID in the population and also of the study inclusion and exclusion criteria, however, size of the cohort is comparable to other CVID microbiome studies. Since previous studies showed that the microbiome of CVID patients is affected by the disease diagnosis, disease severity (11, 13), and also by sharing the same household (13, 14), we have selected paired samples that could minimize the effect of environment and diet, together with selection of CVID patients without gastroenterological symptomatology for minimization of the impact of CVID complications and treatment regiments.

### CVID Microbiota Shows Subtle Differences on a Species Level

In this study, the microbiome composition of CVID patients was, for the first time, analyzed at a species level. More bacterial species were found in CVID patients compared to corresponding controls. The increased microbial diversity was found across the cohort of CVID patients despite their various immunological characteristics, length of treatment, and age. An enriched microbiome has been reported in patients suffering from depression (33), autism (34), atherosclerosis (35), and also in a few studies of colorectal carcinoma (36, 37). Similarly to CVID, atherosclerosis and colorectal carcinoma are conditions with possible alteration of immune functions (38, 39). Increased diversity indicates alteration of the microbial composition that is clearly different from the outgrowth of a few pathobionts, which would result in a decreased diversity as it has been observed for other pathological conditions (40).

However, in our study, the effect of diagnosis on bacterial composition was lower than the effect of the environment and nutrition suggesting that the microbial composition is impacted by several additional factors and highlighting the importance of picking the closest control possible. Recently, two studies used matched housemates as controls for CVID and both observed significant effect of household (13, 14).

In this study, the CVID microbiome showed significantly changed abundance of 34 bacterial species compared to microbiome of housemates. Among CVID patients, we found enriched species, which are frequently associated with various gastrointestinal conditions (e.g., *Bilophila wadsworthia* from *Proteobacteria*) (41), but also species considered beneficial, such as *Alistipes onderdonkii* (*Bacteroidetes*) (42) and *Roseburia* sp. CAG18 (*Clostridia*) (43). Moreover, *Hungatella hathewayi* [previously *Clostridium hathewayi* (44)] was significantly associated with CVID, as it was found among five out of six patients without presence in controls. This is in accordance with Jørgensen et al. (45), who observed the enrichment of genus *Hungatella* in 16S rRNA microbiome of CVID patients. *H. hathewayi* is a part of the normal human intestinal microflora (44, 46, 47), but it is also involved in various human infections (48–50), and its abundance was increased among patients suffering from cystic fibrosis (51), primary IgA nephropathy (52), chronic kidney disease (53), and colon cancer (54–56).

On higher taxonomic levels than species, we did not observe microbial enrichment in CVID, which agrees with the previous 16S rRNA microbiome studies showing that CVID patients, when selecting those without complications, did not differ in α-diversity/richness from healthy controls (11, 13). Moreover, a metagenomic analysis by Fadlallah et al. (15) did not find differences between controls and patients with selective IgA deficiency, which is likely a result of the fact that selective IgA deficiency could be considered a very mild, frequently asymptomatic, form of CVID and because the authors did not use controls living in the same household. On the other hand, Berbers et al. (40) showed enrichment of oropharyngeal microbiota in CVID patients and its association with severity of the immunodeficiency; however, this finding cannot be directly extrapolated to the fecal microbiome without additional experimental support. Although we did not observe significant differences in relative abundance of bacterial phyla between CVID and control samples, a trend for expansion of *Proteobacteria* (5.19% *vs.* 1.49%, q=0.092) among patients was observed (**Figure S2**), which is in accordance with increase of *Proteobacteria* taxa in previous studies (11, 13). Moreover, the expansion of *Proteobacteria* could correspond to fact that IgA immunity preferentially targets *Proteobacteria*, e.g., *E. coli* (15, 57).

Unlike in mild forms of CVID, a reduction of intestinal richness was found in severe CVID conditions with complications (11, 13). Since we found increased diversity of species also in a patient with more severe CVID phenotype but no gastroenterological symptomatology, we hypothesize that microbial expansion comes from immunodeficient conditions, but it appears to be diminished in severe CVID phenotype as a result of diarrhea, enteropathy, and other related complications or treatment regiments. Reduced microbiome diversity is considered a possible universal biomarker of common intestinal diseases, such as inflammatory bowel diseases, colorectal carcinoma, diabetes, and obesity (58), although other studies consider reduced richness as poor marker of dysbiosis (59–61).

Besides identified differences in bacterial compositions, we also observed a trend for increased abundance of *Nematoda* and *Ascomycota* among CVID patients (CVID/control ratio: 6.86 and 2.32, respectively; **Table S4**), which indicate an improper immune response to worms and molds. However, similarly to Fiedorová et al. (13), we did not observe significant alteration of the CVID mycobiome.

### CVID Microbiome Is Enriched for Genes of Nonessential Functions

In accordance with the observed trend for enrichment of species in CVID patients, the CVID microbiota showed even more significant differences on the level of genetic functions. Moreover, a diagnosis was more important for functional composition than the household. In the only related study, published so far, on selective IgA deficiency, Fadlallah et al. (15) did not find difference in a number of identified genes (and also species) among patients and healthy controls.

In this study, the metagenome of CVID patients was enriched for a set of low-abundance genes, likely representing various pathways of nonessential functions. The most significantly altered were genetic functions related to bacterial motility and chemotaxis, whose abundances were increased in CVID patients. This could be potentially explained by the deficiency of IgA immunity, since flagellin is a microbial surface antigen recognized by secretory IgA, which could promote physical clearance of the bacteria from the intestine and/or reduce frequency of motility in bacterial populations (10, 62, 63). The expansion of flagellar bacteria could be related to the enrichment of various bacterial strains/populations inside commensal species, or with the expansion of a few flagellar pathobionts/pathogens, such as motile clostridia (e.g., *Hungatella*, *Roseburia*, and *Anaeromassilibacillus*) and *Proteobacteria*.

### Differences in CVID Metabolome

In this study, CVID patients showed dysregulation in metabolic pathways and decreased levels of several metabolites.

CVID patients showed decreased levels of adenosine and inosine, two metabolites of purine metabolism, which are important anti-inflammatory signal molecules modulating the immune response (64, 65). A decreased level of “purine-like” molecules was previously detected in blood of CVID patients (66). We also observed a decreased abundance of purine-related pathways in the CVID metagenome (**Table S5**), which indicates not only agreement between metagenome and metabolome data, but also microbiome-related alteration in purinergic signaling in the CVID phenotype. It is known that intestinal microbiota also play an important role in metabolic diseases with dysregulation of purine metabolism, such as hyperuricemia and gout (67, 68). In addition, the decreased levels of adenosine resulted in dysregulation of betaine metabolism (**Figure 4A**), which is in accordance with observed changes of plasma level of betaine, a precursor of TMAO (triethylamine N-oxide), among CVID patients in Norway (69). Same authors also observed a positive correlation between levels of *Hungatella* sp. and choline TMA-lyase (*Cut*C) in feces.

Among other functions, bile acids are signal molecules modulating the immune response (70, 71) and we observed deceased levels of glycocholic and glycoursodeoxycholic acid in stool of CVID patients. Dysregulation of bile acid metabolism related to bacterial dysbiosis has been reported for several conditions (72, 73) including a CVID murine model (32). Frequent CVID complications, such as malabsorbtion and/or liver diseases (74–76), also indicate dysregulation of bile acid metabolism. In fact, splenomegaly was frequent among our CVID patients. Interestingly, Li et al. (77) recently described a positive correlation between the presence of *H. hathewayi* and levels of taurine, a part of bile acid metabolism. Both abundances of *H. hathewayi* and the pathway for taurine utilization were enriched in CVID patients.

Dysregulation of vitamin B6 (pyridoxine) metabolism with decreased levels of pyridoxine in stool of CVID patients was observed. Vitamin B6 is cofactor of various enzymes (78), but can also affect the host immune response (79). Moreover, Bierwirth et al. (79) observed decreased plasma levels of pyridoxine among a cohort of CVID patients and suggested that vitamin B6-depletion is consequence of immunodeficiency, probably related to malabsorption.

Taken together, we observed decreased levels of several immunomodulating molecules, which were associated with diagnosis across the cohort of patients. In addition, eight identified metabolites, which stool levels clearly distinguished between CVID patients and controls, could represent indirect markers of disease, which may have a diagnostic and/or prognostic potential, mainly for intestinal complications of CVID.

### Limitations of This Study

This study performed a multi-level analysis of intestinal composition of microbiota in conditions of CVID. A major limitation of this study is the relatively small sample set reflecting the low occurrence of CVID in population together with limited availability of paired samples from healthy housemates and excluding the enrollment of patients with gastrointestinal complications and/or with treatment regiments (i.e., antibiotics, immunosuppression). Another limitation of this study is actual insufficiency of metagenomic and metabolomic databases, which resulted in a limited number of identified species, genetic functions, and metabolites and also a limited assessment of the correlation between metagenome and metabolome.

### Summary

Despite the above-mentioned limitations, this study shows a detailed intestinal CVID microbiome composition with minimalized effect of environment and CVID-related intestinal complications. We have identified increased bacterial diversity in conditions of host immunodeficiency, especially at the level of bacterial species and encoded variable nonessential gene functions. In addition, several bacterial species and stool metabolites were shown to have levels consistently associated with CVID conditions across the cohort of patients with various immunological characteristics, length of treatment, and age. These are potential CVID markers, however, their relevance has to be confirmed on larger cohorts and in functional studies.

While the clinical relevance of the presented results is limited at this moment, this study revealed findings that help understand the relationship between microbiota and host immunity. Since composition of gut microbiota appears to play a fundamental role in host health and disease, identified microbiome features associated with CVID immunodeficiency in this study open new opportunities for further studies with larger cohorts that could potentially address better disease management.

## Data Availability Statement

The datasets presented in this study can be found in online repositories. The names of the repository/repositories and accession number(s) can be found in the article. The data are available under BioProject ID: PRJNA666684 (NCBI database; https://dataview.ncbi.nlm.nih.gov/object/PRJNA666684).

## Ethics Statement

The studies involving human participants were reviewed and approved by the Ethic Committee of the Faculty of Medicine, Masaryk University (Protocol no. 37/2016). The patients/participants provided their written informed consent to participate in this study. Written informed consent was obtained from the individual(s) for the publication of any potentially identifiable images or data included in this article.

## Author Contributions

JL recruited the patients and collected the clinical data. KF and LM prepared the biospecimens. ML performed the data mining and biostatistical analysis. ZS and DG performed metabolomic analysis, JB and DS designed the study, analyzed the data, and wrote the manuscript. JB wrote the first draft of the manuscript. JL, ML, KF, LM, ZS, DG, and TF. contributed to the writing and review of the manuscript. All authors contributed to the article and approved the submitted version.

## Funding

The study was financed by the following funding bodies: Masaryk University (MUNI/M/1322/2015 (DŠ), ROZV/23/LF13/2019 (JB), and ROZV/28/LF/2020 (JB)), Ministry of Education, Youth and Sports (MUNI/A/1099/2019 (KF, TF, JL)), the Grant Agency of the Czech Republic (17-24592Y (ZS)), and the RECETOX research infrastructure: the Czech Ministry of Education, Youth, and Sports (LM2018121, 02.1.01/0.0/0.0/18_046/0015975, and CZ.02.1.01/0.0/0.0/16_013/0001761) and Operational Programme Research, Development and Innovation - project CETOCOEN EXCELLENCE (No CZ.02.1.01/0.0/0.0/17_043/0009632 and 857560).

## Conflict of Interest

The authors declare that the research was conducted in the absence of any commercial or financial relationships that could be construed as a potential conflict of interest.
