# Supplementary material for: Patients With Common Variable Immunodeficiency (CVID) Show Higher Gut Bacterial Diversity and Levels of Low-Abundance Genes Than the Healthy Housemates
Source: Front Immunol. 2021 May 14;12:671239. doi: 10.3389/fimmu.2021.671239 (PMC8163231; doi:10.3389/fimmu.2021.671239)
Supplement: Supplementary file 1 [file DataSheet_1.pdf]

Supplementary Figures

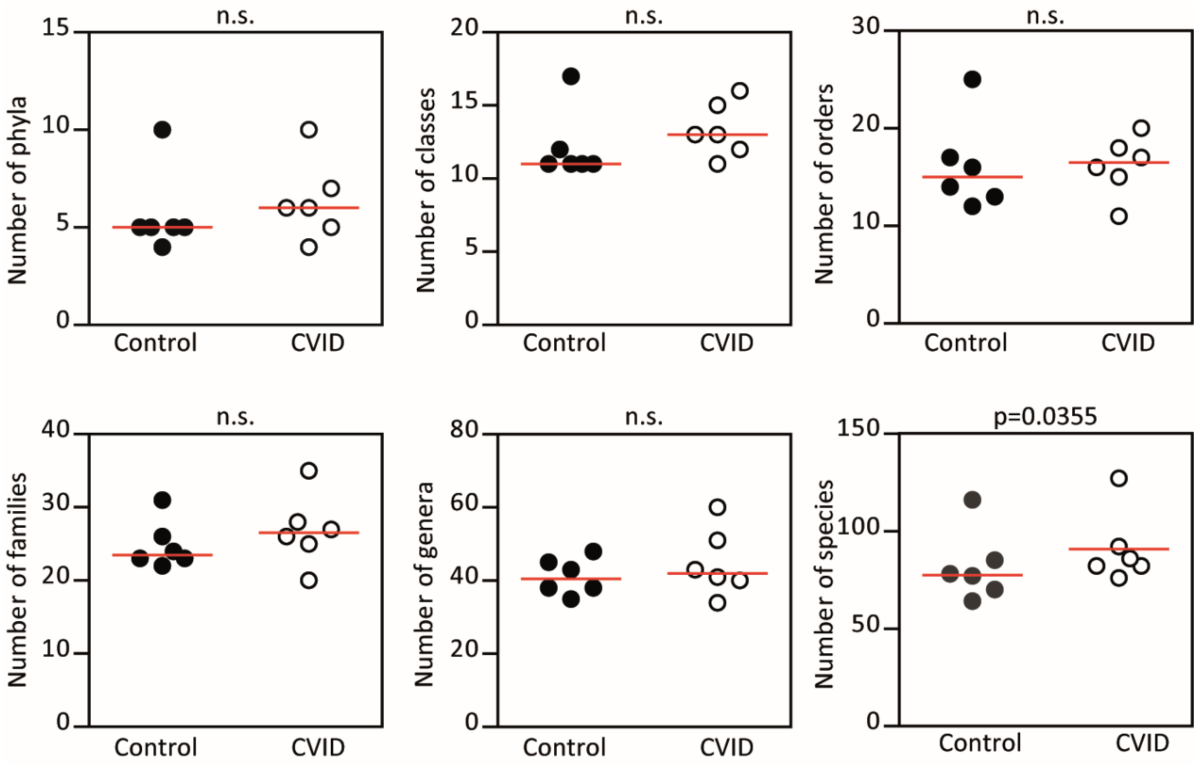

Figure S1: Microbial richness **(A)** and diversity **(B)** at various taxonomic and functional levels. The bacterial richness was similar between CVID patients and healthy controls, except for an increased number of bacterial species in CVID patients. In addition to subtle increase of bacterial species, functional diversity showed significantly different composition (i.e., increased Shannon index). Paired Wilcoxon test was used for statistical analysis. n.s., not significant. Symbols, individuals. Red bar, median. Detailed analysis of diversity is shown in Table S3.

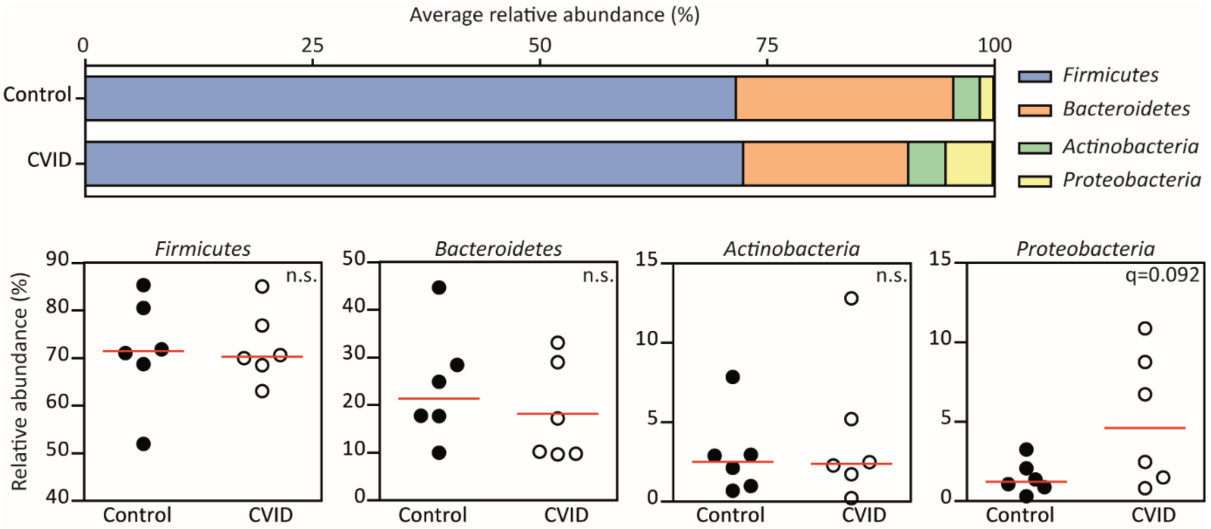

Figure S2: General microbiome composition among CVID patients and healthy controls. CVID patients and controls did not significantly differ in relative abundance of main phyla using DESeq2 analysis (q<0.05), however, we observed trend for reduction of *Bacteroidetes* and expansion of *Proteobacteria*. Only phyla with relative abundance over 1% are presented; and, at the same time, the rest of the present phyla together represent less than 1%. n.s., not significant. Symbols, individuals. Red bar, median.
