# Supplementary material for: Patients With Common Variable Immunodeficiency (CVID) Show Higher Gut Bacterial Diversity and Levels of Low-Abundance Genes Than the Healthy Housemates
Source: Front Immunol. 2021 May 14;12:671239. doi: 10.3389/fimmu.2021.671239 (PMC8163231; doi:10.3389/fimmu.2021.671239)
Supplement: Supplementary file 2 [file DataSheet_2.docx]

### ordination.R

# A script used to calculate distances between samples and make ordination plots

library(vegan)

library(phyloseq)

library(ggplot2)

library(plyr)

# Clear variables from previous calculations, if needed

remove(physeq)

remove(physeq_otu)

remove(physeq_data)

remove(motu)

# Uses abundance data in mspecies matrix object derived from abundance tables

cspecies <- read.table("**Comparison-species-summed-count.txt**", stringsAsFactors=F)

colnames(cspecies) <- c("species","PID002","PID010","PID011","PID015","PID035","PID043","PID003","PID009","PID012","PID004","PID036","PID048")

mspecies <- as.matrix(cspecies[,2:13])

rownames(mspecies) <- cspecies$species

motu <- otu_table(mspecies, taxa_are_rows = TRUE)

sample_df <- data.frame(colnames(cspecies[2:13]),c("C","C","C","C","C","C","P","P","P","P","P","P"), c(1,2,3,4,5,6,1,2,3,4,5,6), stringsAsFactors=FALSE)

colnames(sample_df) <- c("id","treatment","pair")

# next line: simplify rownames if imported rownames have e.g. duplicates

rownames(motu) <- 1:length(rownames(motu))

physeq_otu <- phyloseq(motu)

physeq_data <- phyloseq(sample_data(sample_df))

rownames(physeq_data) <- sample_names(physeq_otu)

physeq <- merge_phyloseq(physeq_otu,physeq_data)

dist_methods <- unlist(distanceMethodList)

dist_methods <- dist_methods[-(1:3)]

dist_methods = dist_methods[-which(dist_methods=="ANY")]

dist_methods <- dist_methods[-2]

my_dist_methods <- dist_methods[c(2,4,7,11,14)]

plist <- vector("list", length(my_dist_methods))

names(plist) = my_dist_methods

for( i in my_dist_methods ){

# Calculate distance matrix

show(i)

iDist <- distance(t(otu_table(physeq)), method=i) # for NMDS

# Calculate ordination

iNMDS <- scores(metaMDS(t(otu_table(physeq)), distance=i, try=100, trymax=1000, autotransform=TRUE))

iNMDS$vectors <- iNMDS

colnames(iNMDS$vectors) <- c("Axis.1","Axis.2")

attr(iNMDS,"class") <- "pcoa"

# Make plot (don't carry over previous plot, if error, p will be blank)

p <- NULL

# Create plot, store as temp variable, p

p <- plot_ordination(physeq, iNMDS, color="pair", shape="treatment")

# Add title to each plot

p <- p + ggtitle(paste("NMDS using distance method ", i, sep=""))

# Save the graphic to file.

plist[[i]] = p

}

# Plot two differently colored version of the NMDS ordination plots

df = ldply(plist, function(x) x$data)

names(df)[1] <- "distance"

p = ggplot(df, aes(Axis.1, Axis.2, color=factor(pair), shape=treatment))

p = p + geom_point(size=3, alpha=0.5)

p = p + facet_wrap(~distance, scales="free")

p = p + ggtitle("NMDS on various distance metrics for CVID dataset")

p

p = ggplot(df, aes(Axis.1, Axis.2, color=treatment))

p = p + geom_point(size=3, alpha=0.5)

p = p + facet_wrap(~distance, scales="free")

p = p + ggtitle("NMDS on various distance metrics for CVID dataset")

p

### clustering.R

# A script to create dendrograms based on alpha diversity

library(phyloseq)

motu <- otu_table(mspecies, taxa_are_rows = TRUE)
sample_df <- data.frame(c("PID002" ,PID010","PID011","PID015","PID035","PID043","PID003","PID009","PID012","PID004","PID036","PID048"),c("C","C","C","C","C","C","P","P","P","P","P","P"), c(1,3,4,6,15,19,1,3,4,6,15,19), stringsAsFactors=FALSE)

colnames(sample_df) <- c("id","treatment","pair")
rownames(motu) <- 1:length(rownames(motu))
physeq_otu <- phyloseq(motu)
physeq_data <- phyloseq(sample_data(sample_df))
rownames(physeq_data) <- sample_names(physeq_otu)
physeq <- merge_phyloseq(physeq_otu,physeq_data)
estimate_richness(physeq)

# A script to create dendrograms based on beta diversity

library(phyloseq)

# physeq variable calculated in ordination.R

plot(hclust(distance(physeq, method="chao")), xlab="Sample dendrogram (using Chao distance matrix", axes=FALSE)

### deseq2.R

# A script to find differentially abundant taxons or functions, based on methodology used with RNA-seq data

library(SummarizedExperiment)

library(DESeq2)

# read in data from abundance table as in aldex2.R and reorder/rename columns

testip <- **cseed2**[,c(1,2,6,7,9,10,12,3,5,8,4,11,13)]

nrows <- length(testip[,1])

ncols <- 12

# reformat to matrix, set sample names

counts <- as.matrix(testip[,2:13])

rownames(counts) <- rownames(testip)

sampleNames <- colnames(testip)[2:13]

sampleTreatment<-c("C","C","C","C","C","C","P","P","P","P","P","P")

colData<-data.frame(sampleName=sampleNames, treatment=sampleTreatment)

# run DESeq2 test

se<-SummarizedExperiment(assays=list(counts=counts), colData=colData, rowData=testip[,1])

dds <- DESeqDataSet(se, design = ~ treatment)

dds <- DESeq(dds)

res <- results(dds, contrast=c("treatment","C","P"))

cbind(testip[,1],res)

write.table(cbind(testip[,1],res),file="**seed2.tab**", sep=",")

### aldex2.R

# A script to calculate significantly overrepresented or underrepresented taxons or functions based on the ALDEx2 methodology

library(ALDEx2)

# read in abundance table from MEGAN

cseed2 <- read.table("**Comparison-seed2-summed-count.txt**", stringsAsFactors=F)

colnames(cseed2) <- c("cseed2","PID002","PID003","PID004","PID009","PID010","PID011","PID012","PID015","PID035","PID036","PID043","PID048")

# reorder columns by diagnosis

test <- cseed2[c(1,2,6,7,9,10,12,3,5,8,4,11,13)]

conds <- c("C","C","C","C","C","C","P","P","P","P","P","P")

# calculate aldex2 test

# denom=”iqlr” used in functional data, mc.samples set to 3000 in the biggest sets SEED3-4)

x <- aldex.clr(test[,2:13], conds, mc.samples=30000, denom="iqlr", verbose=TRUE)

# denom=”all” used in taxonomic data)

#x <- aldex.clr(test[,2:13], conds, mc.samples=30000, denom="all", verbose=TRUE)

x.tt <- aldex.ttest(x, conds, paired.test=TRUE)

x.effect <- aldex.effect(x, conds, include.sample.summary=FALSE, verbose=TRUE, useMC=FALSE)

x.all <- data.frame(x.tt,x.effect)

# plot test results

par(mfrow=c(1,2))

aldex.plot(x.all, type="MA", test="welch")

aldex.plot(x.all, type="MW", test="welch")

# write out a table with values for the labelled points

write.table(cbind(test[,1],x.all), file="**aldex_seed2_Sep_paired.tsv**", sep="\t")

- **NOTE:** Phrases marked in **bold** are specific settings depending on which taxon or function are analyzed, the file naming scheme.
